# Manipulation of *Auxin Response Factor 19* affects seed size in the woody perennial *Jatropha curcas*

**DOI:** 10.1038/srep40844

**Published:** 2017-01-19

**Authors:** Yanwei Sun, Chunming Wang, Ning Wang, Xiyuan Jiang, Huizhu Mao, Changxiang Zhu, Fujiang Wen, Xianghua Wang, Zhijun Lu, Genhua Yue, Zengfu Xu, Jian Ye

**Affiliations:** 1 State Key Laboratory of Plant Genomics, Institute of Microbiology, Chinese Academy of Sciences, 100101, Beijing, China; 2 Temasek Life Sciences Laboratory, National University of Singapore, 117604, Singapore; 3 State Key Laboratory of Crop Genetics and Germplasm Enhancement, Nanjing Agricultural University, 210095, Nanjing, China; 4 Jiangsu Collaborative Innovation Center for Modern Crop Production, China; 5 State Key Laboratory of Crop Biology, Shandong Key Laboratory of Crop Biology, Shandong Agricultural University, 271018, Tai’an, China; 6 Biomass Energy Research Institute, Neijiang Academy of Agricultural Sciences, Sichuan, China; 7 Beijing Plant Protection Station, 100029, Beijing, China; 8 Xishuangbanna Tropical Botanical Garden, Chinese academy of science, China

## Abstract

Seed size is a major determinant of seed yield but few is known about the genetics controlling of seed size in plants. Phytohormones cytokinin and brassinosteroid were known to be involved in the regulation of herbaceous plant seed development. Here we identified a homolog of *Auxin Response Factor 19 (JcARF19*) from a woody plant *Jatropha curcas* and genetically demonstrated its functions in controlling seed size and seed yield. Through Virus Induced Gene Silencing (VIGS), we found that *JcARF1*9 was a positive upstream modulator in auxin signaling and may control plant organ size in *J. curcas*. Importantly, transgenic overexpression of *JcARF19* significantly increased seed size and seed yield in plants *Arabidopsis thaliana* and *J. curcas*, indicating the importance of auxin pathway in seed yield controlling in dicot plants. Transcripts analysis indicated that ectopic expression of *JcARF19 in J. curcas* upregulated auxin responsive genes encoding essential regulators in cell differentiation and cytoskeletal dynamics of seed development. Our data suggested the potential of improving seed traits by precisely engineering auxin signaling in woody perennial plants.

Plant seeds and seed products comprise more than 70% of calories and 60% of all proteins consumed by the human population. Global seed-derived food security is now facing serious problems caused by multiple factors, such as continuous population increase, reduced arable land, global climate change, and the demands for the production of biofuels^1^. The United States Department of Energy estimates that global demand for energy will increase by approximately 35% between 2005 and 2030. Use of sustainable biofuels as energy is predicted to be a major contributor to the increase and a further threat to global food security. The use of agricultural plants such as corn and soybean as feedstock for large-scale biofuel production would also conflict with food production, causing food supply shortages, increased food prices and ethical conflicts^2^.One potential solution to the food crisis is to produce biofuels from plant species capable of growing in marginal lands. Such species should be easy to grow and propagate with high oil yield, and their cultivation should be environmentally beneficial or neutral. Life cycle assessment (LCA) is a quantitative approach to estimating the environmental sustainability of biofuels^3^. However, the widespread commercialization of biodiesel is limited by low seed yields, which drive up costs.

Seed yield is related to seed size, which is predominantly determined by genetic factors. Genetic studies with the model species *Arabidopsis thaliana* and rice (*Oryza sativa*) have identified a number of genes affecting seed size^1^,^4^. Current evidences suggest that seed size is mainly controlled by epigenetics and by genetic pathways, including proteasomal degradation, phytohormones, G protein signaling and small RNA regulation^1^,^4^,^5^,^6^,^7^,^8^,^9^. Although manipulation of these processes could maximize seed yield, how they are regulated and integrated is poorly understood. Furthermore, as a consequence of the complex organization of seeds, only a few studies report yield improvements by direct engineering of genes for either seed size or final seed yield^10^. Therefore, it is necessary to identify more genes from model or non-model plants to understand the genetic network that controls seed size^11^.

Plant hormones play pivotal roles in the developmental processes of diverse traits related to yield^12^. For example, the phytohormones cytokinin and brassinosteroid are involved in the regulation of herbaceous plant seed development^1^. Auxin regulates cell division and is further involved in almost every process of plant growth and development, including seed size. Auxin signaling initiates from hormone perception by an F-box protein receptor Transport Inhibitor Response 1 (TIR1) followed by degradation of negative regulators AUX/IAA proteins^13^. Auxin Response Factors (ARFs) are then released. In the current auxin signaling model, Aux/IAAs dimerize with and repress ARF transcription factors in the absence of auxin. In the presence of auxin, Aux/IAAs interact with TIR1 resulting in repressor degradation, freeing ARFs for auxin-responsive gene transcription^14^. The interaction between ARFs and IAAs is a key aspect of auxin regulation and occurs through the highly conserved COOH-terminal (CT) Phox and Bem1p (PB1) domain present in both types of proteins^15^,^16^,^17^,^18^.Genetically, auxin induces the formation of Arabidopsis pluripotent cells via a root development pathway by *ARF7, ARF19* and other downstream transcription factors such as Lateral Organ Boundaries-domain (LBD) *LBD16, LBD17, LBD18* and *LBD29*^19^. They bind to auxin-responsive elements (AuxREs) in promoter regions to enhance or repress auxin-regulated genes and auxin-inducible genes. In *Arabidopsis, ARF2* negatively regulates auxin signaling and seed size via downregulation of cell division in the integument region of endosperm. However, *arf2* plants exhibit pleiotropic effects, e.g. reduced seed set, which make these plants undesirable for agriculture^20^,^21^,^22^. Rice *THOUSAND-GRAIN WEIGHT 6 (TGW6*) encodes an IAA-glucosehydrolase and plays an essential role in the regulation of auxin homeostasis during endosperm development. Downregulation of this negative regulator in the auxin pathway increases grain weight and yield in rice^23^. Activation of the *BIG GRAIN1 (BG1*) gene, which encodes a regulator in auxin transporting in rice, leads to significantly increased seed size and plant biomass, seed weight and yield^24^. The *YUCCA1 (ZmYUC1*) gene in maize (*Zea mays*) encodes a seed-specific flavin monooxygenase that is involved in tryptophan-dependent IAA biosynthesis; its mutant has 40% less dry mass than wild-type seeds^25^. However, which ARFs and how the ARFs and AUX/IAA proteins control seed development remains largely unknown^12^.

Compared with conventional herbaceous vegetative oil, seed oil from perennial woody plants has notable nutritional advantages as well as potential raw materials for biodiesel production. Woody plants are usually perennials with strong secondary xylem that can efficiently transport water and nutrients from root to leaf. *Jatropha curcas (Jatropha*) is one promising new energy crop with high seed oil content, tolerance to drought and an ability to thrive in poor soil^26^. Furthermore, the reduction of green house gas emission for generating 1 gigajoule energy can be at least 40–107% with respect to fossil diesel^27^,^28^,^29^. However, *Jatropha* has some undesirable traits, such as a high male-flower ratio, high sensitivity to viral diseases, high polyunsaturated fatty acid level and low seed yields^30^,^31^,^32^. To improve the inadequacy, biotechnological progress had been made in the seed oil profile, flowering traits and virus resistance of *Jatropha*^31^,^33^,^34^,^35^,^36^, however, seed size and seed yield of *Jatropha* cultivars would need to improve to meet seed production demands^34^. It is known that auxin plays an important role in the growth of *Jatropha*. Pretreated cuttings *Jatropha* with indole-3-butyric acid (IBA) and 1-naphthalene acetic acid (NAA) increased rooting, sprouting, and seeds yield. Auxin pretreated cuttings produced much longer and healthier plants that flowered and produced fruits and seeds a year earlier than the control^37^. However the detailed molecular mechanism how extrogenous application of auxin can increase seed yield is still unknown.

Seed size in *J. curcas* is greater than in the congener *J. integerrima* (Fig. 1a). Using the inter-species crossing population *J. curcas* × *J. integerrima*, we conducted a whole-genome scan for quantitative trait loci (QTLs) and expression QTLs that affect seed oil traits in *Jatropha*^11^,^38^,^39^. We screened key genes in auxin pathway including *ARFs, IAAs* and downstream effectors to identify candidate genes controlling seed size. Importantly, we detected a major QTL *qSL11-a* controlling seed length with a high likelihood of odd score (LOD score) of 16.69 and percentage of variance explained (PVE) 29.6% on Linkage group (LG) 11, were harboring the *JcARF19* gene^11^.

In this study, we functionally characterized a key genetic factor *ARF19* which regulates seed size and seed yield in *Jatropha*. We found that *JcARF1*9 functions as a positive upstream modulator. Its ectopic expression leads to increase in cell size and cell number in *Jatropha*. Transgenic expression of *JcARF19* can also increase seed size and seed weight both in *Arabidopsis* and *Jatropha*.

## Results

### Down regulation of *JcARF19* affected auxin signalling transduction in *Jatropha*

**Figure 1:**
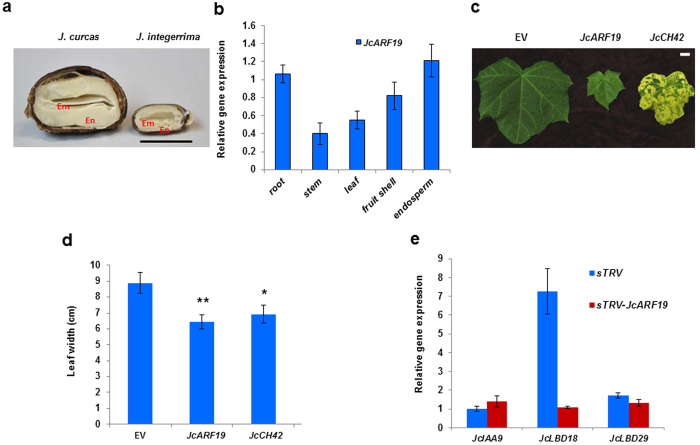
Auxin responses in *Jatropha curcas* leaves of silenced *JcARF19*. (**a**) Seed cross-sections of *J. curcas* (left) and *J. integerrima* (right). En: Endosperm; Em: Embryo. Size bar: 10 mm. (**b**) Expression of *JcARF19* in different organs. Root, stem and leaf samples were harvested at six weeks after fertilization and fruit shell and endosperm samples were collected at five weeks after fertilization. Each organ had three biological replicates and each biological replicate with three technical replicates. Values (n = 3) were shown as mean ± SD. The relative expression level of *JcARF19* in *Jatropha* was normalized with the expression level of 18S RNA in *Jatropha*. (**c**) Phenotypes of *Jatropha* plants leaves at 27 days post-infiltration (dpi) with various synthetic TRV (sTRV) constructs: Empty vector (EV), sTRV: *JcARF19 (JcARF19)*, sTRV: *JcCH42 (JcCH42*). Bar: 10 mm. (**d**) The width of leaves from *Jatropha* plants infiltrated with various synthetic TRV constructs at 27 dpi. Values (n = 5) were shown as mean ± SD and statistic analysis with Student T-test. **Indicates P < 0.01. *Indicates P < 0.05. (**e**) Relative expression levels of *JcLBD18* and *JcLBD29* in systemic leaves of plants infiltrated with sTRV empty vector (EV) and sTRV: *JcARF19 (JcARF19*). Samples were collected after treatment of IAA (10 nM) and each treatment had three biological replicates. For qRT-PCR, each biological replicate was replicated three times. Numbers represent mean relative values from three independent experiments with standard deviation. The relative expression level of *JcIAA9* in sTRV- silenced plants was normalized as 1.

According to the whole-genome scan results, we hypothesized that *JcARF19* might participate in controlling seed size. To test the hypothesis, firstly, we investigated potential tissue-specific roles of *JcARF19* in *Jatropha* by profiling its expression patterns in roots, stems, leaves, fruits and seeds using quantitative real-time PCR. *JcARF19* showed highly similar expression profiles to ubiquitous expression in all organs and highest in endosperm (Fig. 1b), providing evidence that *JcARF19* might be related to seed traits in *Jatropha*. To test the function of *JcARF19* in auxin signalling transduction and therefore plant development in *Jatropha*, we used synthetic tobacco rattle virus (sTRV) based virus-induced gene silencing (VIGS) method, which we developed previously and allowed us to rapidly identify gene function in various plants^40^,^41^. At 27 days after inoculation of agrobacterium containing VIGS vectors of *JcARF19* and positive control *Jatropha Chlorata 42 (JcCH42*) or empty vector control (EV), we observed distinct smaller newly expanding leaves in *JcARF19-*silenced plants than EV-treated plants (Fig. 1c) and the difference between them was remarkably as shown in Fig. 1d^34^. Smaller leaf size was also observed in the positive control treatment containing the silenced marker gene, *JcCH42*, which encodes a subunit of Magnesium (Mg) chelatase, involved in photosynthesis (Fig. 1c)^42^. Recent research showed that auxin treatment enhances ARF19 binding to its target gene promoters, which correlates with the enhancement of transcriptional activity of the ARF19 in *Arabidoposis thaliana (Arabidopsis*)^43^.We next tested the gene expression of downstream transcription factors esp. Lateral Organ Boundaries-domain (LBDs). Upon auxin treatment, two genes *JcLBD18* and *JcLBD29* which encode putative LBDs of *Jatropha* were up-regulated differentially (Fig. 1e), especially *JcLBD18* which had 7-fold higher expression in EV-treated plants. But in *JcARF19-*silenced plants, there was no obvious induction of *JcLBD18* or *JcLBD29* upon auxin treatment, indicating that *JcARF19* was essential for proper auxin-mediated signalling transduction process in leaf cells of *Jatropha*.

### Transgenic overexpression of *JcARF19* increased seed size and dry seed weight in *Arabidopsis*

**Figure 2:**
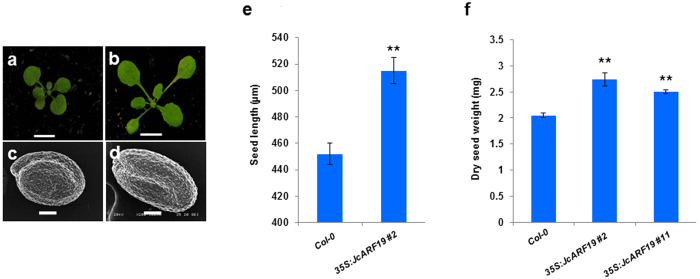
Increase of seed size and seed weight by overexpression of *JcARF19* in *Arabidopsis*. Fourteen-day-old WT Columbia ecotype (Col-0) *Arabidopsis* (**a**) and *35S:JcARF19* overexpression plants (**b**). T3 generation plants were used for observations. Bar: 1 cm. SEM observation seeds of WT Col-0 (**c**) and *35S:JcARF19* overexpression plants (**d**). T3 generation plant seeds were used for observations. Bar: 100 μm. (**e**) Seed length of WT Col-0 and *35S: JcARF19* overexpression *Arabidopsis*. Values are mean ± SD (n = 10) and statistic analysis with Student T-test. **Indicates *P* < 0.01. (**f**) Dry seed weight of 100 seeds of WT Col-0 and *35S: JcARF19* overexpression *Arabidopsis*. Values are mean ± SD (n = 10) and statistic analysis with Student T-test. **Indicates *P* < 0.01.

To further investigate the function of *JcARF19*, at first, we got partial cDNA sequence from a database of sequenced cDNA library prepared from *Jatropha* seeds^11^. By integrating known genomic sequence of *JcARF19* and cloned sequenced information from 5′RACE and 3′RACE, we finally got the full-length coding sequence of *JcARF19* (Genbank accession NO. KX988008, detailed sequence information could be found in Supplementary file). JcARF19 protein encoded 1133 amino acids and consists of major functional domains, an amine-terminus B3 DNA binding domain and a carboxyl-terminus (CT) Phox and Bem1p (PB1) domain. Amino acid sequence alignment showed that ARF19s from various plants had high sequence similarity in the three conserved domains (Fig. S1), suggesting functional conservation of ARF19 family proteins. To further investigated the function of JcARF19, we generated thirty transgenic lines for *CaMV35S:JcARF19 in Arabidoposis*^44^. An obvious increase in plant size and seed size was observed in the *35S:JcARF19* plants compared to wild-type (WT) Col-0 plants (Fig. 2a–d). Seed length and seed dry weight of *35S:JcARF19* lines were increased remarkably compared with those of WT Col-0 plants as well (Fig. 2e,f). We further conducted oil traits analysis to check the effects of *JcARF19* on oil yield or oil composition. There was no significant change in either oil content per dry seed weight or oil composition in transgenic line of *35S:JcARF19* compared with those of WT Col-0 plants (Fig. S2). Since seed size and weight were significantly increased in transgenic line of *35S:JcARF19*, the lipid content per seed was obviously increased. These results indicated that overexpression of *JcARF19* increased seed size, seed dry weight and oil yield, but had no effect on oil composition of seeds in *Arabidopsis*.

### Increased seed size and seed numbers by overexpression of *JcARF19* in *Jatropha*

**Figure 3:**
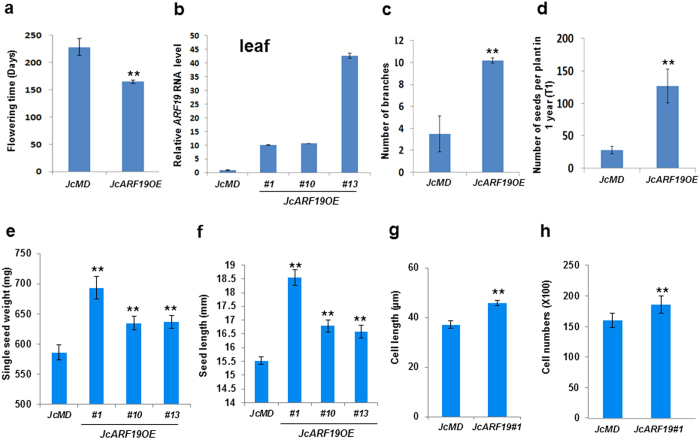
Agronomic traits of *JcARF19*-overexpressing *Jatropha*. (**a**) Flowering time in 10 independent T0 transgenic *Jatropha* plants overexpressing *JcARF19* and wild-type plant *Jc*-MD. Values are mean ± SD (n = 10), and Student T-test statistic was used for analysis. **Indicates *P* < 0.01, the same for the below traits analysis. (**b**) Relative *JcARF19* expression level in leaves of T0 generation *35S:JcARF19* transgenic *Jatropha* lines (*JcARF19OE#1, #10* and *#13*) and wild-type plant *Jc*-MD. Values are mean ± SD (n = 3). (**c**) Comparison of branch number of WT plant *Jc*-MD and ten T0 *JcARF19* overexpression lines. Values are mean ± SEM (n = 10). (**d**) Comparison of seed number per tree per year of WT plant *Jc*-MD and ten T0 *JcARF19OE* lines. Values are mean ± SEM (n = 10). (**e**) Comparison of single seed weight of WT plant *Jc*-MD and three T0 *JcARF19* overexpression (*JcARF19OE #1, #10* and *#13*) line. Values are mean ± SEM (n = 50). (**f**) Comparison of seed length of WT plant *Jc*-MD and three *JcARF19OE #1, #10* and *#13* lines. Values are mean ± SEM (n = 50). (**g**) Comparison of cell length of WT plant *Jc*-MD and *JcARF19OE #1* line. Values are mean ± SEM (n = 10). (**h**) Comparison of seed cell number of WT plant *Jc*-MD and *JcARF1*9OE #1 line. Values are mean ± SEM (n = 10).

**Figure 4:**
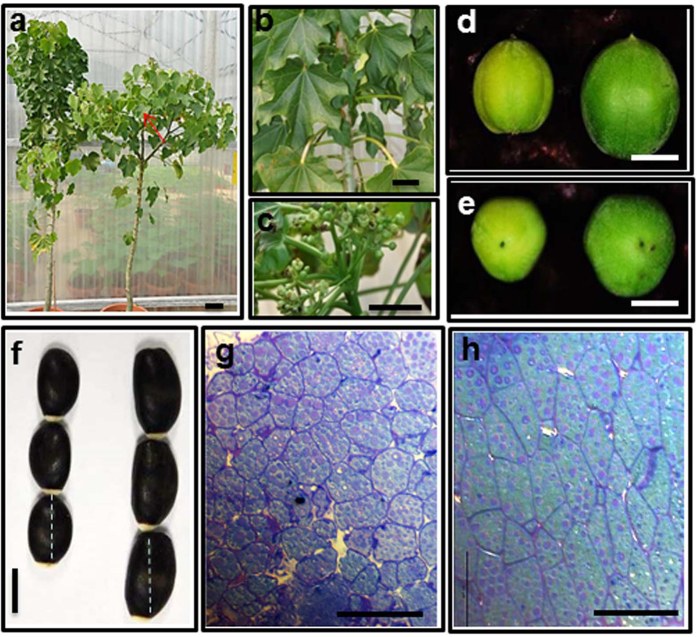
Functional analysis of *JcARF19* in *Jatropha.* (**a**) Comparison of plant architecture of *JcARF19* overexpression transgenic T0 plant (#1, right) with WT *Jatropha* plant (*Jc*-MD, left). (**b**) No inflorescence was found in WT *Jatropha* plant *Jc*-MD in the same stage as *JcARF19*-overexpressing transgenic T0 plant (#1). (**c**) *JcARF19-*overexpressing transgenic T0 plant (#1) with early inflorescence. Bar: 10 cm for (**a**–**c**). (**d,e**) Comparison of fruit size of WT *Jatropha (Jc*-MD, left) and transgenic T1 Jatropha plant overexpressing *JcARF19* (right). Bar: 1 cm. (**f**) Comparison of seed size of WT *Jatropha (Jc*-MD, left) and transgenic T1 *Jatropha* plant overexpressing *JcARF19* (right). Dashed line indicates the position of the cross-section. Bar: 1 cm. Comparison of cell size of WT Jatropha Jc-MD (**g**) and transgenic T1 Jatropha plant overexpressing *JcARF19* (**h**). Bar: 50 μm.

*Jatropha* was also transformed with *35S:JcARF19* vector^31^. The ectopic expression of *JcARF19* affected calluses formation during transformation procedure (Fig. S3), producing bigger calluses than that of empty vector (EV). After transferring to soil, the ectopic expression of *JcARF19* also affected flowering time (Fig. 3a) and other characteristics (Fig. 3c–f). Under normal growth conditions in a greenhouse, WT *Jatropha Jc*-MD required around 8 months to produce the first inflorescence whereas 10 primary independent *JcARF19* overexpression lines formed their first inflorescences after only 5 months, with a 3 months reduction (Figs 3a and 4a–c). Quantitative reverse transcriptase PCR (qRT-PCR) analysis verified the presence of the transgene and ectopic expression of *JcARF19* in transgenic *Jatropha* plants (Fig. 3b). *JcARF19* overexpression lines also had greater branching compared with WT *Jc*-MD control plants (Figs 3c and 4a). A four-fold increase in seed set from *JcARF19* ectopic expression plants was collected within one year of transplanting. Furthermore, a 17.2% increase of single seed weight and 17.1% increase in fruit size were found in *JcARF19* ectopic expression plants (Figs 3d–f and 4d–f). The longer cell length (18.4%) and higher cell number (16.5%) in *JcARF19* ectopic expression plants explained the increased seed weights and lengths (Figs 3g,h and 4g,h). These results indicated that overexpression of *JcARF19* increased length, number and weight of seeds and also oil yield by increasing seed cell number and length in *Jatropha*.

We further germinated T1 *JcARF19-*overexpression *Jatropha* seeds and found that the germination percentage of T1 *JcARF19*-overexpression *Jatropha* seeds was higher than these of the wild type control (Fig. S4).

### Expression of Auxin responsible genes upon the ectopic expression of *JcARF19* in developing seeds

**Figure 5:**
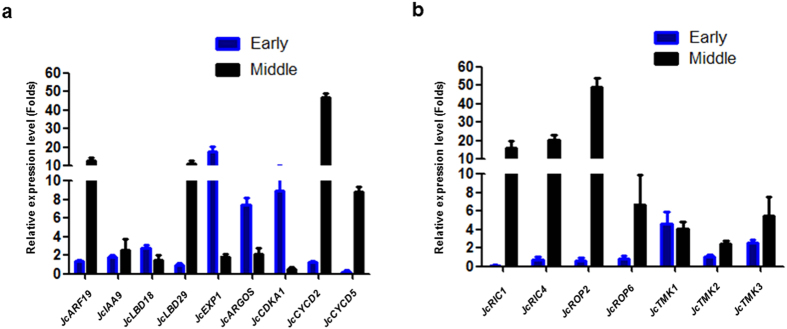
Relative expression in developing endosperm of the *JcARF19* overexpression plants. Early stage: 3WAF, Middle stage: 6WAF. Values are mean ± SD (n = 3). (**a**) Relative expression folds of cell cycle and cell number related genes (*JcARF19, JcIAA9, JcLBD18, JcLBD29, JcEXP1, JcARGOS, JcCDKA1, JcCYCD2* and *JcCYCD5*) in *JcARF19* #1 overexpression plants normalized with the wild type. (**b**) Relative expression folds of cell differentiation and cytoskeletal dynamics related genes (*JcRIC1, JcRIC4, JcROP2, JcROP6, JcTMK1, JcTMK2* and *JcTMK3*) in *JcARF19* #1 overexpression plants normalized with the wild type.

In VIGS assay, we have demonstrated that *JcARF19* is essential for auxin signaling transduction on downstream transcription factors *JcLBD18* and *JcLBD29* in *Jatropha* leaves (Fig. 1e). To further understand its role in auxin pathway during seed developing, we analyzed the expression of *JcLBD18* and *JcLBD29* in *JcARF19* overexpression seeds and found that both of two *LBD* genes were higher expressed either at early stage or middle stage compared with those of WT control. We further checked the expression of other downstream genes in auxin pathway such as cell cycle and cell number controlling. We found that the expression level of *Expansin1 (JcEXP1*) and *AUXIN-REGULATED GENE INVOLVED IN ORGAN SIZE (JcARGOS*)^45^, together with cell cycle regulators *JcCDKA1, JcCYCD2* and *JcCYCD5*, are induced in *JcARF19*-ectopic expression *Jatropha* seeds endosperm, at either early or middle stage of seed development (Fig. 5a), providing molecular explanations for the increased cell number in *JcARF19* overexpression *Jatropha* seeds. We found that the expression of several genes encoding important regulators in cell differentiation and cytoskeletal dynamics have been enhanced including ARGOS^46^, small GTPases auxin-Rho of Plants (ROP), ROP-interactive protein RIC and receptor-like auxin-(Transmembrane Kinase) TMK^47^,^48^, either in early or middle stages of seed development of *J. curcas* (Fig. 5b), which also explain the cell size expansion in *JcARF19* ectopic expression *Jatropha* seeds.

### Characterization of interaction between JcARF19 and JcIAA9

**Figure 6:**
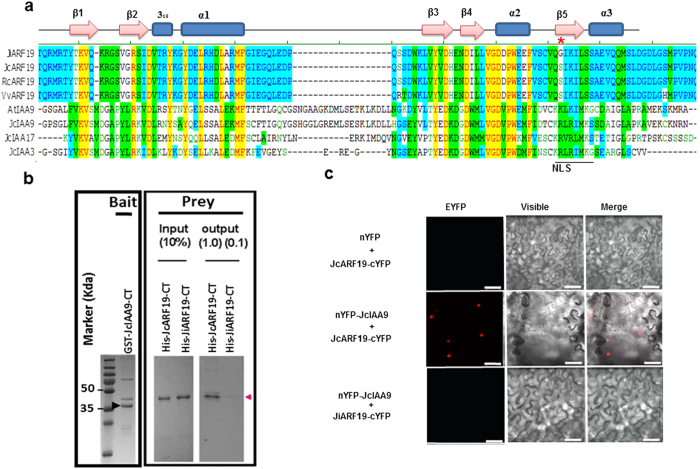
The key animo acid residue affected the ability of JcARF19 binding with JcIAA9. (**a**) Predicated secondary structure of JcIAA9, JcARF19 and other related proteins. (**b**) GST pull-down assay. Recombinant bait protein GST-JcIAA9 COOH-terminal (residues 229–368 shows JcIAA9*-*CT containing protein-protein interaction domain III and domain IV of Aux/IAA protein family) strongly binds to prey protein JcARF19 COOH-terminal (residues 1005–1276), but weakly binds to prey protein JiARF19-CT (residues 1008–1279). (**c**) BiFC assay. Very strong fluorescence signal was only found in the combination of C-terminal of JcIAA9 and JcARF19 (nYFP-JcIAA9+JcARF19-cYFP), but not in the combination of C-terminal of JcIAA9 and JiARF19 (nYFP-JcIAA9+JiARF19-cYFP). Bar: 50 μm.

Each ARF transcription factor forms dimer with corresponding Aux/IAAs. Stimulated by eQTL clue of the genetic interaction of *JcARF19* and *JcIAA9* we identified previously, we hypothesized that ARF19 may function via direct protein-protein interaction with IAA9. The secondary structure of JcARF19 and JcIAA9 were predicted to have 4 α-helix and 5 β-sheet folding module (Fig. 6a). We found that JcARF19 interacts with JcIAA9 physically *in vitro* pull-down assays (Fig. 6b). We used glutathione S-transferase (GST) fused JcIAA9 as bait and JcARF19 as prey. We found that the GST-fused JcIAA9 COOH-terminal protein (JcIAA9-CT) could interact strongly with 6*Histidine-tagged ARF19 COOH-terminal protein (JcARF19-CT), in contrast to much weaker interaction found on the protein pair of J. integerrima (JiARF19 and JcIAA9). This difference was surprising because the ARF19-PB1 and IAA9-PB1 polypeptides of the two species differ by only one amino acid. The S → G mutation which located in interaction interface β5 of ARF19 proteins affects ARF19-IAA9 binding ability. We also provided evidence to show the physical interaction between JcARF19 and JcIAA9 *in vivo* and the vital role of key amino acid in heterodimer formation by Bimolecular Fluorescence Complementation (BiFC) assays (Fig. 6c). However, although we identified a putative protein-protein interaction pair of IAA9-ARF19 here (Fig. 6), it is still unclear what the significance of this interaction is so far and how IAA9 affects the function of ARF19 in auxin signaling pathway.

## Discussion

In herbaceous plants, species with small seeds sometimes have larger seed set than larger-seed species, assuming a limited total amount of energy. This energetic trade-off has been observed in genetic mutants such as *APETALA2*^49^,^50^,^51^ and *CURLY LEAF28*^52^. In this study we showed that manipulation of the auxin pathway in *J.curcas* not only increased seed size but also enhanced total seed yield. Previous knowledge on the molecular mechanisms of seed size was mainly limited to model herbaceous plants, particularly *Arabidopsis* and rice^53^. Our work suggests that manipulation of auxin is an alternative approach to increase seed size in woody plants.

Several lines of evidence support the involvement of *JcARF19* in seed size determination. First, *JcARF19* was mapped in the major quantitative trait locus (QTL) region and was significantly associated with seed size^11^. Second, by using expression QTL (eQTL) analysis to link variants with functional candidate genes, we provided evidences that seed traits were affected by the genetic interaction of *JcARF19* and *JcIAA9*^11^. Third, the C-terminal of JcARF19, which is essential for protein-protein interaction among ARF proteins, was identical between ARF19 homologs of bigger seed size in *Jatropha*. Point mutations on the single-nucleotide polymorphisms (SNPs) of *JcARF19* affected their direct physical interactions (Fig. 6). Fourth, overexpression of *JcARF19* increased seed size in both *Arabidopsis* and *Jatropha* by increasing both cell numbers and cell length. Fifth, ectopic expression of *JcARF19* upregulated auxin responsive genes encoding important regulators involving in cell differentiation and cytoskeletal dynamics.

Woody plants such as *Jatropha* had longer life cycle than herbaceous ones. It took as long as two years for *Jatropha* to get the first flower blooming under our current lab condition in Beijing North of China, on contrast of 4–5 months in tropical countries such as Singapore. We tried to plant *Jatropha* in South of China and so far they do not flower yet. For this reason we used a few independent T0 *Jatropha* plants and we also performed genetic analyses on laboratory model plant *Arabidopsis*. The increased seeds yield of T3 *JcARF19* ectopic *Arabidopsis* was also consist with the results of overexpression in *Jatropha*, confirming our claims of improved seed traits with *JcARF19* ectopic expression strategy. Furthermore, we found that the germination percentage of T1 *JcARF19-*overexpression *Jatropha* seeds was obviously higher than that of the wild type (Fig. S4). Early research had reported that the auxin has close relationship with the seeds germination rate^54^. It indicated that the traits we observed at T0 generation can be inherited into the next generation and our improved seed traits by *JcARF19* ectopic expression are reliable as well^55^. Nevertheless, the observed impact on plant architecture, seed size and yield by manipulation of *ARF19* need not only more observations on T1 and T2 generations plants under greenhouse condition, more researches under field condition are also necessary for the feasibility of a big scale commercialization of this strategy. Auxin is a multifunctional hormone that regulates pattern formation in plants^56^. The location and timing of auxin accumulation and signal transduction play critical roles in various aspects of plant development^57^,^58^. In the future, to avoid growth abnormalities in auxin signaling pathway transgenic plants, it is advisable to use a weaker or an organ and developmental specific promoter rather than a stronger promoter like *CaMV 35S* promoter because the amount of the hormone produced by the transgene and the response should be confined to the target tissue at an appropriate level as did in cotton fiber cell and other success reports^59^.

We present a common sharing genetic framework for the control of cell division, differentiation and size for various plant organs, e.g. seed and root. Given the early working model of *ARF19* in auxin signaling transduction^53^,^55^,^60^, the seed size controlling results in this study can be best integrated as the working model presented in Fig. S5. In this seed size controlling model, auxin activates the transcription of *JcARF19* via RETINOBLASTOMA-RELATED (RBR) protein and cytokinin-dependent transcription factor *ARABIDOPSIS RESPONSE REGULATOR12 (ARR12*)^60^. *JcARF19* is involved in promoting cell differentiation and thus cell number increasing in early stage of seed development by regulating the transcription of *LBD18* and *LBD29*^55^. *ARGOS* gene family is auxin-induced and involved in the regulation of cell number for the duration of organ growth^45^. Ectopic expression of ARGOS prolongs the expression of AINTEGUMENTA (ANT) and cell cycle regulator CycD3; as well as the neoplastic activity of leaf cells^45^. Overexpression of ARGOS genes modifies plant sensitivity to Ethylene, leading to improved drought tolerance in both *Arabidopsis* and maize^46^. The auxin-(Transmembrane Kinase) TMK sensing and auxin-Rho of Plants (ROP) signaling networks have been demonstrated to control auxin signaling pathway^47^,^48^. *JcARF19* might be also involved in enlarged cell size by TMK Auxin-Sensing and ROP GTPase signaling complex in middle stage of seed development. Considering that orthologs of *JcARF19* exist in many other plant species, including castor bean, alfalfa (*Medicago sativa*), soybean and apple, the manipulation of *ARF19* may provide a broad application to increase plant biomass and seed productivity in many other species.This study provides evidence that an auxin signaling integrator ARF19 plays vital roles in determining seed size. ARF19 is conserved in higher plants and involved in auxin pathway signal transduction^55^. Nevertheless, it is necessary to test the *ARF19* ectopic expression *Jatropha* lines under field conditions to get conclusive statements of its commercial viability. Meanwhile besides of plant genome, plant rhizospheric or leaf-residing microbiomes via plant endogenous auxin signalling pathway have been successfully to improve crop yield dramatically including *Jatropha*^61^,^62^. *ARF19* transcription factors mediated auxin pathway is essential for growth and yield promoting effect by beneficial microbes^63^. Manipulation of the auxin signaling pathway can result in larger seed sizes and improved seed yield in *J. curcas,* this *ARF19*-ectopic expression plant may become a more attractive commercial plant.

## Materials and Methods

### Plant materials and growth condition

Three species of plants were used in this study. For *Jatropha*, seeds were obtained from the *Jatropha curcas (Jc*-MD) elite plants which were pre-selected by Drs. Yan Hong and Chenxin Yi^64^. The seeds were germinated on ½ Murashige and Skoog salt medium at 25 °C under a 16 h light/8 h dark photoperiod with a light intensity of 100 μmol·m^−2^ s^−1^. When two or three true leaves were grown^64^, the seedlings were transplanted into pots filled with soil and grown at 25 °C in greenhouse under natural light condition. Plant management, including pesticide spraying, watering and artificial fertilization, was carried out according to normal practice^31^. For *Arabidopsis*, seeds of ecotype Col-0 were vernalized on ½ Murashige and Skoog salt medium at 4 °C in darkness for about three days and transferred into a growth chamber at 22 °C under16 h dark/8 h light photoperiod. The seedling with four true leaves were transplanted into soil and grown in the same condition. *Arabidopsis* transformation was performed according to the floral-dipping method previously described^65^. For *Nicotiana benthamiana*, seeds were sown in soil to germinate. The seedlings were transplanted in separated pots and grown in a greenhouse at 25 °C with 14 h dark/10 h light photoperiod.

### RNA extraction and analysis

RNA was isolated and analyzed according to previously described methods^23^. 100 mg samples from different organs were harvested and extracted with plant RNA purification reagent (Invitrogen, Carlsbad, CA, USA). Nanodrop (Thermo Scientific, Wilmington, DE, USA) was used to quantify RNA concentration. M-MLV reverse transcriptase (Promega, Madison, USA) was used for reverse transcription. For quantitative PCR analysis, THUNDERBIRD SYBR qPCR Mix (TOYOBO) was used and run in Bio-Rad CFX96 qPCR machine. Each treatment was repeated with three biological replicates, and with three technical replicates for each biological sample. The *Jatropha UBQ* transcript was served as an internal control for RNA samples. The primers for target genes are listed in Table S1. Standard deviation was calculated based on the three biological replicates.

### Virus induced gene silencing

We used the sTRV method described by Ye *et al*.^40^,^41^, using psTRV1 and psTRV2. PCR-based cloning was used to clone partial cDNAs of *JcARF19* to psTRV2 to generate psTRV2 derivatives. Another psTRV2 clone with insertion of *Jatropha Chlorata 42 (JcCH42*) was served as a positive control^34^. psTRV1, psTRV2 and psTRV2 derivatives were electroporated into *Agrobacterium* strain AGL1. Vacuum agroinfiltration was used to inoculate those *Agrobacterium* into *Jatropha* seedling with two or three true leaves. At least 5 *Jatropha* seedlings were agroinfiltrated with psTRV1 and psTRV2-*JcARF19*, psTRV2-*JcCH42* or psTRV2 vector only accordingly. After infiltration, plants were grown in a growth chamber at 25 °C with a 16 h light ⁄8 h dark photoperiod^40^. Phenotypes of *Jatropha* plants at 27 days post-infiltration (dpi) with various sTRV constructs were recorded and leaves in same leaf position were picked and leaf width were measured. Values (n = 5) were shown as mean ± SD and statistic analysis with Student T-test. **Indicates *P* < 0.01, *indicates *P* < 0.05.

### IAA sensitivity test

Detached silenced systemic *Jatropha* leaves were kept in liquid MS medium containing 10 nM IAA following treated with vacuum infiltration. Treated leaves were incubated at 25 °C in an incubator for 6 hrs. After the IAA treatment, the leaves were subjected to RNA isolation and following quantitative real-time PCR analysis.

### Transgenic plant plasmid construction

*JcARF19* gene was identified from a database of sequenced cDNA library prepared from *Jatropha* seeds (detailed sequence information could be found in Supplementary file). The full-length cDNA fragment of *JcARF19* was PCR-amplified with primers (Table S1). The PCR fragment was inserted in the sense orientation into suitable sites of pCABMIA1300-3HA vector^34^.

### Scanning electronic microscopy (SEM) and light microscopy

For observation of *Arabidopsis* seeds with the scanning electron microscope (SEM), collected seeds from WT Col-0 and *35S:JcARF19* overexpression plants respectively were fixed with a tape inside a sample chamber, following freezing in liquid N2. Images were collected using a SEM (JSM-6360LV, JEOL, USA) with an acceleration voltage of 20 kV. For observation of *Jatropha* seed endosperm with light microscopy, endosperm discs from WT *Jc*-MD and *35S:JcARF19* overexpression plants respectively were excised from mature *Jatropha* endosperm (7 WAF, weeks after fertilization) and fixed overnight in 2.5% glutaraldehyde in 0.1 M phosphate buffer, pH 7.2 as described previously^40^. Endosperm discs were rinsed three times in 0.1 M phosphate buffer for 15 min each, and were then post-fixed in 1% (w/v) aqueous OsO_4_ for 1 h. Tissues were dehydrated in an ethanol series and embedded in Spurr’s resin. Semi-thin sections with thickness of 500 nm were stained in 0.1% toluidine blue and photographed with a Zeiss Axioplan 2 microscope (Carl Zeiss, Germany). Cell size and cell number per disc were analyzed with ImageJ and calculated, followed with statistic analysis with Student T-test. **Indicates *P* < 0.01. Values are mean ± SEM (n = 10).

### Arabidopsis seed size and weight measurement

Mature seeds were harvested from WT Col-0 and *35S:JcARF19* overexpression plants grown under the same conditions. 100 seeds from ten independent transgenic lines and WT Col-0 were weighted and recorded with three technical replicates. Values (n = 10) are given as mean ± SD. A DM5000B microscope (Leica) and ImageJ analysis software were used to measure seed sizes. Values (n = 10) are given as mean ± SD. Statistic analysis with Student T-test. **Indicates *P* < 0.01.

### Fatty acid analysis

Total lipid was extracted and transmethylated from 100 dry *Arabidopsis* seeds as described previously^66^. The resulting FAMEs were separated and detected by GCMS-QP2010 Ultra (Shimadzu, Kyoto, Japan). The GC analysis was performed under conditions described before^31^. The data were presented based on three biological replicates and each biological replicate had three technical replicates. Values (n = 3) are given as means ± standard deviation.

### Explant material preparation and *Jatropha* transformation method

Cotyledons were harvested from WT plants *Jc*-MD sterilized seedlings that were 7–9 days old and were cut into small pieces (5 mm × 5 mm) used as explants. After co-cultivation, shoot regeneration, shoot elongation and rooting, we got the *JcARF19* overexpression line. Detailed protocol can be found in Qu *et al*.^31^.

### *JcARF19*-**overexpressing** *Jatropha* agronomic traits measurement and statistical analysis

Wild-type *Jc*-MD and *JcARF19* transgenic overexpression *Jatropha* plants were grown in the same condition. Flowering time was scored by the number of days from transplantation to soil to the day of first inflorescence emergence. 10 independent T0 *JcARF19* overexpression plants and WT plant *Jc*-MD should be calculated and values are mean ± SD (n = 10). Branch number of each plants after 1 year of transplanting was recorded for either *Jc*-MD (n = 10) or T0 *JcARF19* overexpression plants (n = 10). Values are mean ± SEM (n = 10). Single seed weight and seed length for each seeds were measured for three of WT plants *Jc*-MD and T1 *JcARF19* overexpression seeds of three lines, *JcARF19OE #1, #10* and *#13*. Values are mean ± SEM (n = 50). Germination percentage were measured for five of WT plants *Jc*-MD and T1 *JcARF19* overexpression seeds of *JcARF19* OE #10 and #13. Values are mean ± SEM (n = 5). Student *T*-test was used for statistical analyses for all agronomic traits. **Indicates *P* < 0.01, *indicates *P* < 0.05.

### *In vitro* GST pull-down assay

The C-terminal sequence of *JcIAA9* and *JcARF19* were amplified by PCR using Phusion High-Fidelity DNA Polymerase (Thermo-Fisher, Finnzymes, Espoo, Finnland) and subcloned into pGEX6P-1 or pET28-SUMO vectors to generate GST fusion or 6*His fusion constructs. Point mutations were performed to generate vector of JiARF19 fusion with 6*His tag by QuikChange Site-Directed Mutagenesis Kit (Stratagene, Agilent, Wilmington, DE, USA). *In vitro* pull-down assays were performed with 2 μg of GST fusion proteins and 2 μg of His-tagged proteins. GST fusion proteins were incubated in a binding buffer (50 mM Tris-HCl at pH 7.5, 100 mM NaCl, 0.25% Triton X-100, 35 mM β-mercaptoethanol) with 25 μL of glutathione sepharose 4B (GE Healthcare, Uppsala, Sweden) for 3 h at 4 °C and GST beads were washed six times with binding buffer. His-tagged JcARF19-CT and JiARF19-CT proteins were added into GST beads and the mixture was incubated overnight at 4 °C. After washing again with binding buffer six times, pulled-down proteins were separated on 12% SDS–polyacrylamide gel and detected by Western blotting using anti-His or anti-GST antibody as previously described^67^,^68^,^69^.

### Bimolecular fluorescence complementation (BiFC)

BiFC was carried using previously described vectors and methods^67^,^68^. The C-terminal sequence of *JcARF19* and *JcIAA9* were cloned in corresponding restrict enzyme sites of BiFC vectors. Point mutations were performed to generate *JiARF19* by QuikChange Site-Directed Mutagenesis Kit (Stratagene, Agilent, Wilmington, DE, USA). The resulting cassettes including fusion proteins and constitutive promoters were cloned into pGreen binary vector HY105 and transformed into *Agrobacterium*. For BiFC experiments, 3-week-old *Nicotiana benthamiana* leaves were co-infiltrated with Agrobacterium as previously described. Two days after incubation, fluorescence and DAPI staining were analyzed by confocal microscopy^68^,^70^. The confocal laser scanning microscope technique we used was referred to the Leica SP8 microscope instruction.

## Additional Information

**How to cite this article**: Sun, Y. *et al*. Manipulation of *Auxin Response Factor 19* affects seed size in the woody perennial *Jatropha curcas. Sci. Rep.* **7**, 40844; doi: 10.1038/srep40844 (2017).

**Publisher's note:** Springer Nature remains neutral with regard to jurisdictional claims in published maps and institutional affiliations.

## Supplementary Material

Supplementary Information
