# Supplementary material for: Manipulation of Auxin Response Factor 19 affects seed size in the woody perennial Jatropha curcas
Source: Sci Rep. 2017 Jan 19;7:40844. doi: 10.1038/srep40844 (PMC5244365; doi:10.1038/srep40844)
Supplement: Supplementary Information [file srep40844-s1.pdf]

Supplementary information

Manipulation *of Auxin Response Factor 19* affects seed size in the woody perennial *Jatropha curcas*

Yan-Wei Sun[1, 2#], Chun-Ming Wang[3, 5#], Ning Wang[1, 4#], Xi-Yuan Jiang[2], Hui-Zhu Mao[2], Chang-Xiang Zhu[4] , Fu-Jiang Wen[4], Xiang-Hua Wang[6], Zhi-Jun Lu[7],Gen-Hua Yue[2], Zeng-fu Xu[8], Jian Ye[1,2*]

[1] State Key Laboratory of Plant Genomics, Institute of Microbiology, Chinese Academy of Sciences, 100101, Beijing, China;

[2] Temasek Life Sciences Laboratory, National University of Singapore, 117604Singapore, Singapore;

[3] State Key Laboratory of Crop Genetics and Germplasm Enhancement, Nanjing Agricultural University, 210095, Nanjing, China;

[4] State Key Laboratory of Crop Biology, Shandong Key Laboratory of Crop Biology, Shandong Agricultural University, 271018, Tai'an, China;

[5]Jiangsu Collaborative Innovation Center for Modern Crop Production, China;

[6]Biomass Energy Research Institute, Neijiang Academy of Agricultural Sciences, Sichuan, China;

[7] Beijing Plant Protection Station, 100029, Beijing, China;

[8] Xishuangbanna Tropical Botanical Garden, Chinese academy of science, China.

# Equal contribution,

[*]Correspondence author.

Nucleotide sequence of *JcARF19*, 3402bp

ATGAAGGCTCCTCCAAATGGTTTTATGTCAAATTCTGCAGAAGGAGAAAGGAAGAGTATTAATTCAGAGCTATGGCATGC
ATGTGCGGGGCCGCTGGTCTCTCTGCCTCCAGTTGGAAGTCTTGTAGTTTACTTCCCCCAAGGCCACAGTGAGCAAGTCG
CAGCATCTATGCAAAAGGAGACTGATTTTATACCAAGCTATCCCAACCTTCCTTCCAAGTTGATTTGTATGCTTCATAATGT
CACATTGCATGCTGATGCAGAAACTGATGAGGTCTATGCTCAGATGACCCTTCAACCTGTAAGCAAATATGACAAGGAAG
CATTACTGGCATCTGATATGGGCCTCAAGCAAAGCAGGCAACCTGCCGAATTCTTTTGCAAAACTCTCACAGCTAGTGACA
CAAGTACACATGGTGGATTCTCCGTACCTCGCCGAGCAGCTGAGAAGATTTTCCCACCACTAGATTTTTCAATGCAACCAC
CTGCCCAGGAGCTGGTAGCCAGGGATTTACATGATAATACATGGACATTTAGACATATTTATCGCGGCCAACCAAAACGA
CATCTGCTGACTACAGGTTGGAGTGTGTTTGTCAGCACAAAAAGACTTTTTGCCGGTGATTCTGTTTTATTTATAAGAGAT
GAAAAGTCACAGCTCCTCTTGGGCATTAGGCGTGCTAATAGGCAACAGCCAGCACTCTCTTCGTCAGTCATATCCAGCGA
CAGTATGCATATTGGAATTCTTGCTGCTGCAGCCCATGCTGCTGCAAATAACAGTCCATTTGCTGTATTTTACAATCCAAGG
GCAAGCCCTGCTGAGTTTGTGATTCCCTTCTCCAAGTATAACAAGGCAATGTATACACAAGTTTCACTGGGCATGAGATTC
AGAATGATGTTTGAGACGGAGGAGTCAGGAGTTCGTAGGTACATGGGCACAATCACTGGCATCAGTGATTTGGATCCAG
TGAGGTGGAAAAAATTCACAATGGCGCAATCTTCAGGTTGGATGGGACGAGTCAACTGCTGGTGAACGGCCAAGCCGAGT
TTCAATTTGGGAAATTGAACCCGTTGTAACTCCTTTCTACATTTGTCCACCTCCATTTTTCAGACCCAAGTTCCCTAGACAGC
CAGGGATGCCAGATGATGAGTCTGACATTGAGAATGCTTTTAAAAGGGCCATGCCCTGGCTTGGAGATGATTTTGGCATG
AAAGATTCCCCAAGCTCAATTTTTCCTGGTTTGAGTTTAGTTCAGTGGATGAGCATGCAACAAAATAATCAGTTTCCAGCT
GCTCAAGCAGGATTCTTCCCACCAATGCTTCCTTCCAATGCCTTGCACAATAGCCTTGGCTCTGATGATCCGTCGAAGATAT
TGAATTTTCAAGCACCTGGCCTTTCGGTACCAAGCCTCCAGTTTAACAAAGCGAACCCACAAAACCAAGTCAGCCAGTTGC
CACAGACATCAATGGCATGGACTCAGCAGCAGCAACTGCAGCAATTACTGCAGAGTAATGTCAACCAGCAACAACCATCA
AATCCTCAACAGCAACAGCAGCAACCTCACCCCCAGCAGCAACAACAGAGGCAGCAACCACAACCAACACAGCAACAACT
GAACCAACAACAGCAGCCGCAGCCGCAGCCGCAGCCGCAGCCGCAGCCTCAGCCTCAGCCTCAGCCACAGTCTCAACAA
ATACGACAACAGCCTCCACAATTGCAACTGCAGCAGCAGCAGCAGATATTCCAATCACCACCAGTAAATAATGGTGTGGT
TACCTCTAACCAAATTCCAAATCAAAATTTGCAGCAACCAATTGTATACTCTCAGCTGCAGCAGCAGCAGCTACTGGCAAG
TAATAACCAATCCCAGAACATTCCATCTGCTAATAAGAACTCATATCCACTGACCTCTTTGCCACAAGACCCCCAGTTCCAG
CAACAAATGGAGCAGCAATCTAACCTCTTACAGAGACACCAGCAGCAGACACAGCTGCAACAGTCTCCCCTGCAGTTGTT
GCAACAAAATCTGTCACAGAGGGCACAGCCACAATCACAGTCACAGCCACAAGCGCAACAACTGTCGCAGCCTGGCCTCT
CAGATCAACAGCTTCACTTGCAGTTGCTGCAGAAACTTCAGCAACAACAGCAGCAGCAGCAGCAGCAGCAGTTGCTTTCC
CCTTCAAGCTCACCTTTGCAACCTCAATTGATACAACAGCAGACCCATCAACAAAACCTGCAGTTCCAGCAGTCAACTATTT
CACAGAGCCAGCAGCAACCACTCGGTAGCAACAGCTTCTCAACGGCAGCACTCATGCAATCACAATCTTTTCCTGTCAGCC
AGCACCATGCCCTTCAGAAATCTCCAACAATTAGAGCTCATTCCACTCTTACAGATGGAGATGCTCCATCTTGTTCAACCTC
ACCATCCACTAATAATTGCCAGATTGCACCATCAAATTTCCTGAACAGAAACCAACAAGCACCTGCTATATTGATGGGGGA
TTCAGTGATTGAGCCTGCAACAAATCTTGTTCAAGAGCTCAATAACAAGTCTGATATTCGGGTTAAGCATGAGTTTCCTGG
TTCAAAAGCATCAGACCAACTAAAATACAAAGGTACAATAACAGATCAATTGGAAGCCTCCTCGTCTGGGACATCATATT
GTCTCGATGCTGGAAACATCCAGCAAAATTTCTCACTCCCCACCTTTGGTTTGGATGGTGATGTCCAATCACATCCTCGGA
ACAGTCTGCCTTTTGCAACTAATATTGAGAGCTTGGCGCCTGATACTTTGTTGACAAGGGGATATGACTCTCAAAAAGATC
TTCAAAACTTGCTAGCTAATTATGGTGGGACTCCAAGAGATATTGAGACAGAATTGTCCACTGCTGCAATAAGCTCTCAGT
CATTTGGGGTGCCAATCATGCCCTTCAAGCCTGGATGTTCAAATGATGTTGCAATCAATGACTCTGGGGTTTTGAATGGTG
GATTGTGGGCCAATCAGACTCAACGCATGCGAACATATACAAAGGTTCAAAAGCGTGGCTCAGTGGGCAGGTCTATTGA
TGTCACCCGGTATAAAGGGTATGATGAACTTAGGCATGACCTAGCTCGCATGTTTGGAATTGAAGGGCAACTAGAAGATC
CACAAAGTTCTGACTGGAAGTTAGTTTATGTGGATCACGAAAACGACATACTACTTGTTGGCGATGACCCTTGGGAGGAA
TTTGTTAGCTGTGTTCAGAGCATAAAGATCTTGTCATCTGCCGAAGTGCAACAAATGAGTTTAGATGGTGATCTTGGTAGT
ATGCCAGTTCCAAATCAAGCATGCAGTGGGACTGACAGCGGAAATGCATGGAGAGGACACTATGATGATAACTCAGCTG
CCTCATTTAATAGATAA

Amino acid sequence of *JcARF19*, 1133 aa

MKAPPNGFMSNSAEGERKSINSELWHACAGPLVSLPPVGSLVVYFPQGHSEQVAASMQKETDFIPSYPNLPSKLICM
LHNVTLHADAETDEVYAQMTLQPVSKYDKEALLASDMGLKQSRQPAEFFCKTLTASDTSTHGGFSVPRRAAEKIFPPL
DFSMQPPAQELVARDLHDNTWTFRHIYRGQPKRHLLTTGWSVFVSTKRLFAGDSVLFIRDEKSQLLLGIRRANRQQP
ALSSSVISSDSMHIGILAAAAHAAANNSPFAVFYNPRASPAEFVIPFSKYNKAMYTQVSLGMRFRMMFETEESGVRR
YMGTITGISDLDPVRWKNSQWRNLQVGWDESTAGERPSRVSIWEIEPVVTPFYICPPPFFRPKFPRQPGMPDDESDI
ENAFKRAMPWLGDDFGMKDSPSSIFPGLSLVQWMSMQQNNQFPAAQAGFFPPMLPSNALHNSLGSDDPSKILN
FQAPGLSVPSLQFNKANPQNQVSQLPQTSMAWTQQQQLQQLLQSNVNQQQPSNPQQQQQQPHPQQQQQRQ
QPQPTQQQLNQQQQPQPQPQPQPQPQPQPQPQSQQIRQQPPQLQLQQQQQIFQSPPVNNGVVTSNQIPNQN
LQQPIVYSQLQQQQLLASNNQSQNIPSANKNSYPLTSLPQDPQFQQQMEQQSNLLQRHQQQTQLQQSPLQLLQQ
NLSQRAQPQSQSQPQAQQLSQPGLSDQQLHLQLLQKLQQQQQQQQQQQLLSPSSSPLQPQLIQQQTHQQNLQF
QQSTISQSQQQPLGSNSFSTAALMQSQSFPVSQHHALQKSPTIRAHSTLTDGDAPSCSTSPSTNNCQIAPSNFLNRN
QQAPAILMGDSVIEPATNLVQELNNKSDIRVKHEFPGSKASDQLKYKGTITDQLEASSSGTSYCLDAGNIQQNFSLPTF
GLDGDVQSHPRNSLPFATNIESLAPDTLLTRGYDSQKDLQNLLANYGGTPRDIETELSTAAISSQSFGVPIMPFKPGCS
NDVAINDSGVLNGGLWANQTQRMRTYTKVQKRGSVGRSIDVTRYKGYDELRHDLARMFGIEGQLEDPQSSDWKLV
YVDHENDILLVGDDPWEEFVSCVQSIKILSSAEVQQMSLDGDLGSMPVPNQACSGTDSGNAWRGHYDDNSAASF
NR

Table S1 Primers used in this work

Primer	Sequence (5'-3')	Purpose
JcARF19-Xb5	AATAATCTAGAAATTGCCAGATTGCACCATCA	VIGS
JcARF19-BH3	TGTATGGATCCCAATAGACCTGCCCACTGAG	VIGS
JcARF19-FL-F	ATATAGTCGACATGAAGGCTCCTCCAAATGGT	Plant expression
JcARF19-FL-R	TATATTCTAGATCTATTAAATGAGGCAGCTGAGTT	Plant expression
JcARF19-C-BH5	ATAATGGATCCTTTGCAACTAATATTGAGAGCT	E.coli expression
JcARF19-C-Xh3	TCTATCTCGAGTTATCTATTAAATGAGGCAGCT	E.coli expression
JcIAA9-C-BH5	ATAAAGGATCCCTCGCCACCACATCAAAGAA	E.coli expression
JcIAA9-C-Sal3	TTTATGTCGACCTAGTTCCGGTTCTTACATTT	E.coli expression
JcARF19-PM-F	GTTAGCTGTGTTCAGGGCATAAAGATCTTGTCATCTGC	Point mutation
JcARF19-PM-R	GCAGATGACAAGATCTTTATGCCCTGAACACAGCTAAC	Point mutation
JcARF19-F	GTATGCTTCATAATGTCACATTGCA	Q-PCR
JcARF19-R	CAGGTGCTTGAAAATTCAATATCTT	Q-PCR
JcIAA9-F	GCAATGTTACTTTGCTGGCTTCTT	Q-PCR
JcIAA9-R	AGATTGTGATCCTGGAAGCCCAA	Q-PCR
JcLBD18- F	TGGAAGCAGTAGCAGTGGTG	Q-PCR
JcLBD18-R	CACCGCATCAAGCCTTTTAT	Q-PCR
JcLBD29- F	ACACATTTTGCAGCCATTCA	Q-PCR
JcLBD29-R	ACGGTTTGAGCTGCTTGTTC	Q-PCR
JcEXP1-F	GAGGTGGTGATGCTTCTGGT	Q-PCR
JcEXP1-R	CAGCTGAAGCCATTGTTGAA	Q-PCR
JcARGOS-F	TGGGTTTTGAAGGTCAAAGG	Q-PCR
JcARGOS-R	GAAATCCCCAAAACCCCTAA	Q-PCR
JcRIC1-F	CAGCTAGACGGCATTCATCA	Q-PCR
JcRIC1-R	TTGTGCTTTTGATCGAGACG	Q-PCR
JcRIC4-F	AGATGTGAAGCATGTGACGC	Q-PCR
JcRIC4-R	TGCAGCCATAGAAAGCTCAA	Q-PCR
JcROP2-F	TTTCCTACGGATTACGTGCC	Q-PCR
JcROP2-R	AAGATCGAGCTTTGTTCCGA	Q-PCR
JcROP6-F	GGGTGCAGATGTGTTCCTTT	Q-PCR
JcROP6-R	GGGAATGAACATTTTGCCTG	Q-PCR
JcTMK1-F	TCGGGAAGTAGTGGTGGTTC	Q-PCR
JcTMK1-R	TGCATTTGGACTCTGCACTC	Q-PCR
JcTMK2-F	GATTGAGCATTGCCTTGGAT	Q-PCR
JcTMK2-R	TACTCCGGTGCAAGATACCC	Q-PCR
JcTMK3-F	TTTCCACATCTGCTGCTGAC	Q-PCR
JcTMK3-R	CCACTCCAGCTACAGAAGCC	Q-PCR
JcUBQ-F	GAGGTGGAAAGCTCAGATACAATT	Q-PCR
JcUBQ-R	AAAGTGATGGTCTTTCCGGTCAATG	Q-PCR
JcCDKA1-F	AAGATGAAGGTGTTCCTAGCACT	Q-PCR
JcCDKA1-R	AGCAAATTCAGGACAAGAATCCAT	Q-PCR
JcCYCD2-F	TTCTTGGAATTCAGGCCTTCAGAGA	Q-PCR
JcCYCD2-R	AGCTTCCAACACCCCATTTGGACT	Q-PCR
JcCYCD5-F	GATTCCACACAGCTTATCTTTCAGT	Q-PCR
JcCYCD5-R	GTATTCAATACCAGCAACTCCATT	Q-PCR
Jc18S-F	TATGCTTGTCTCAAAGATTAA	Q-PCR
Jc18S-R	ATAAATGCATCCCTTCCAGAA	Q-PCR

Fig. S1

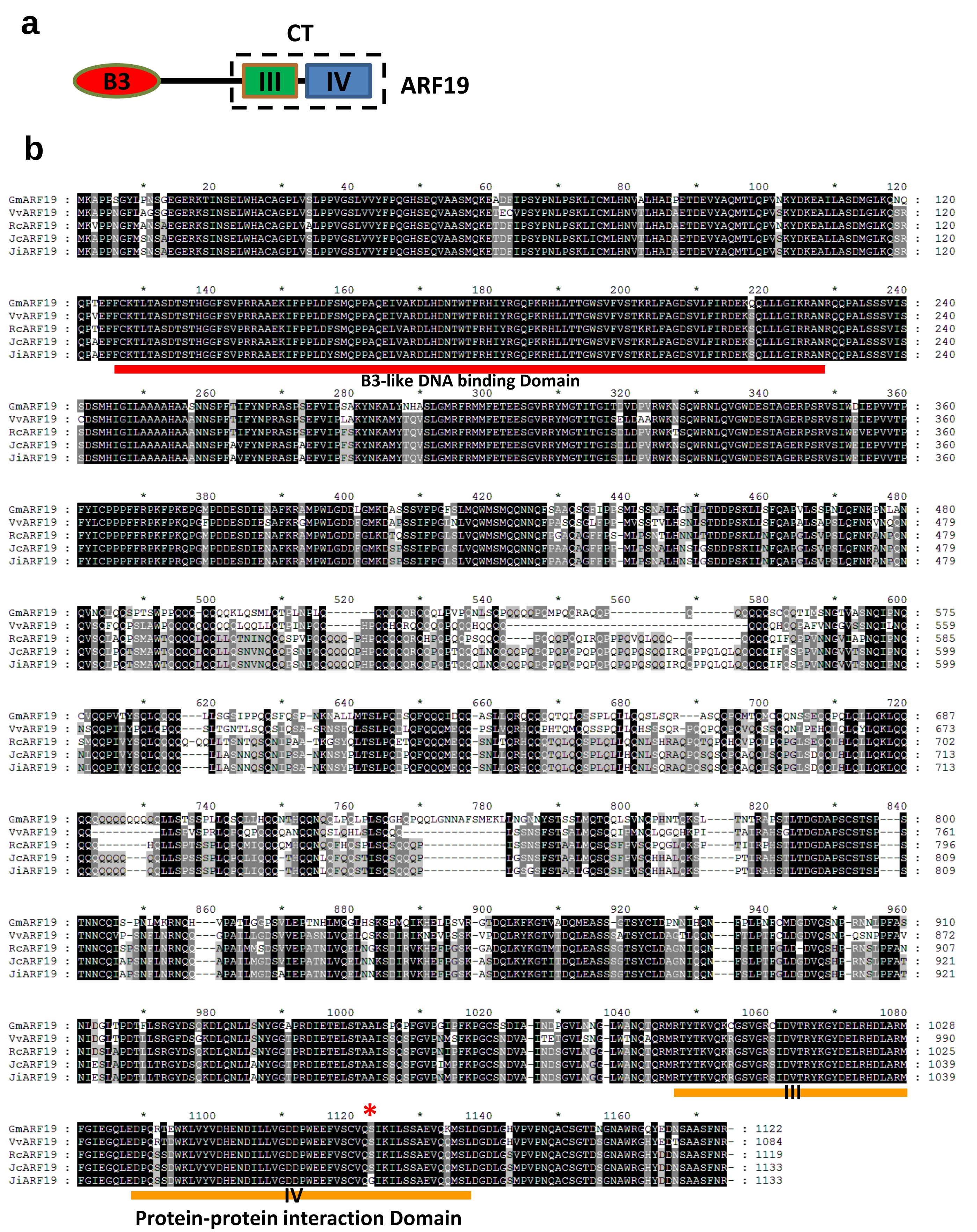

Figure S1 Domain structure (a) and amino acid sequence alignment (b) of ARF19 protein from various plants. Red line indicates for B3-like DNA binding domain. Yellow line indicates the protein-protein interaction domain. The key residue in JcARF19 are indicated by *.

Fig. S2

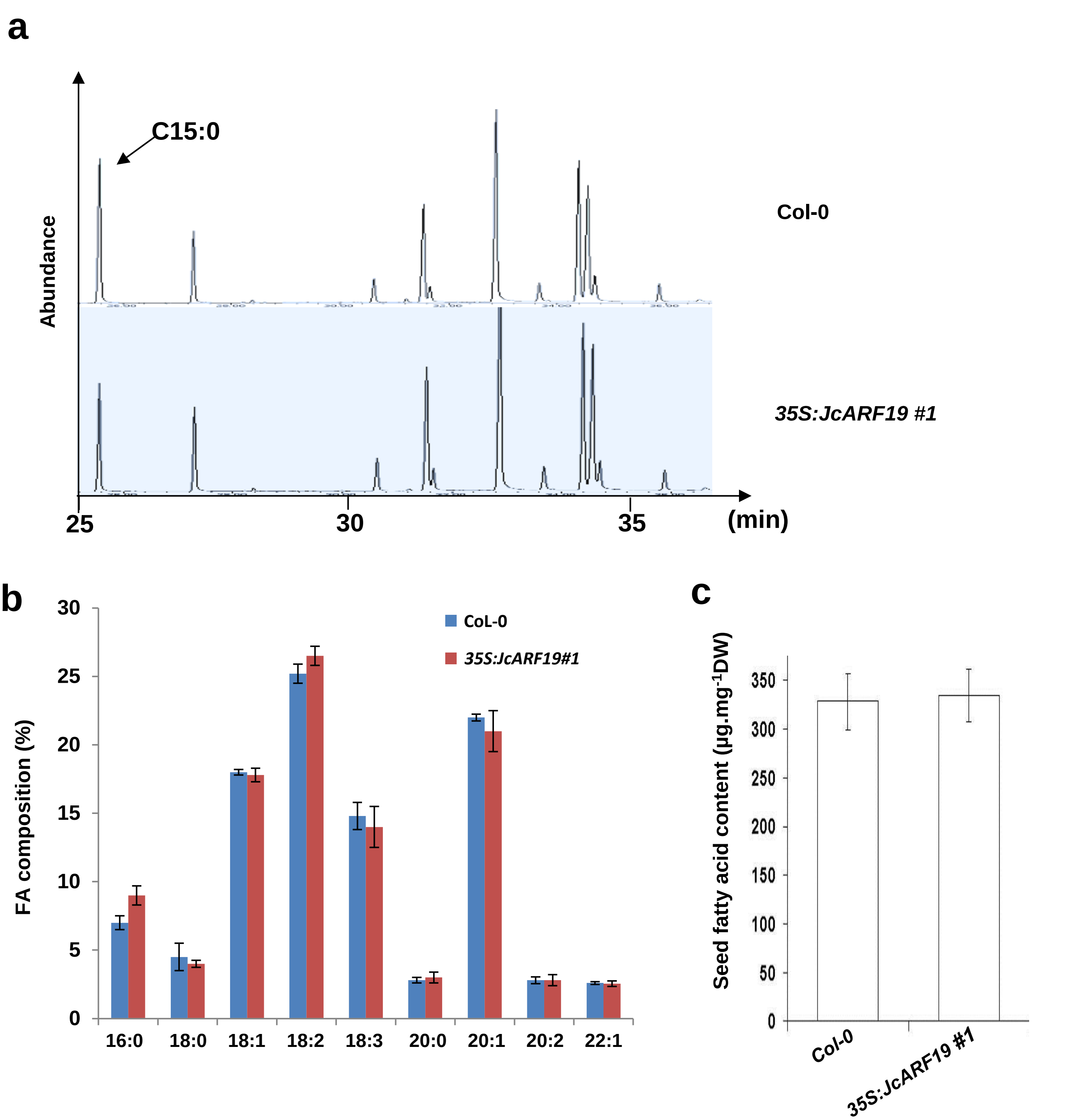

Figure S2 Seed fatty acid composition and content analysis for WT Col-0 and *JcARF19* overexpression *Arabidopsis* plants. Values are mean ± SD (n=3).

a and b. Seed fatty acid composition analysis of WT Col-0 and *JcARF19* overexpression *Arabidopsis* plants. The numbers shown at X-axis in Fig b meant various types of fatty acid tested from WT Col-0 and *JcARF19* overexpression *Arabidopsis* plants. 16:0 meant palmitic acid, 18:0 meant stearic acid, 18:1 meant oleic acid, 18:2 meant linoleic acid, 18:3 meant α-linolenic acid, 20:0 meant arachidic acid , 20:1 meant cis-11-eicosenoic acid, 20:2 meant cis-11,14-eicosadienoic acid , 22:1 meant erucic acid.

c. Seed fatty acid content analysis for WT Col-0 and *JcARF19* overexpression *Arabidopsis* plants.

Fig. S3

a **b**

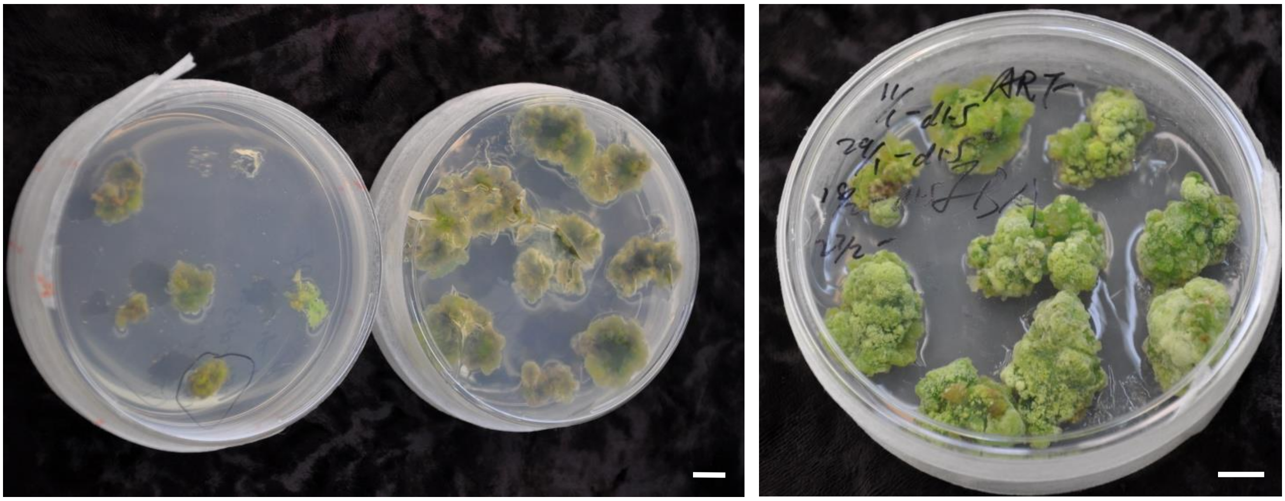

EV 35S:JcARF19 35S:JcARF19

Figure S3 Calluses formation had visible differences between empty vector (EV) control and overexpreesion of *JcARF19* (*35S:JcARF19*). Bar: 1cm.

a. Calluses formation from cotyledon explants treated by agrobacterium containing *35S:JcARF19* (right) compared with empty vector control (EV, left) were rapidly and dramatically enhanced in callus-inducing medium.

b. Enlarged view of the front side of calluses from cotyledon explants treated by agrobacterium containing *35S:JcARF19*.

Fig. S5

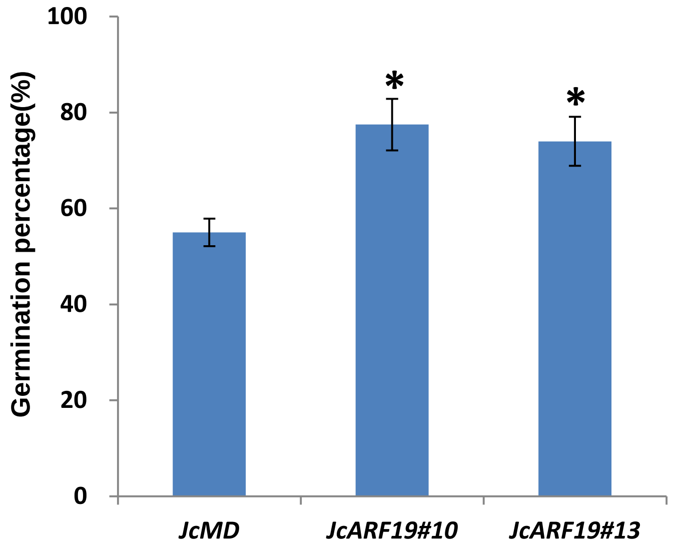

Figure S4 Germination percentage of *JcARF19* T1 overexpression seeds.
Mature seeds were harvested from wild type *Jc*-MD and *JcARF19* overexpression plants and grown under the same condition. Values are mean ± SEM (n =5). Student *T*-test was used for statistical analysis. * indicates $P < 0.05$.

Fig. S5 Working Model

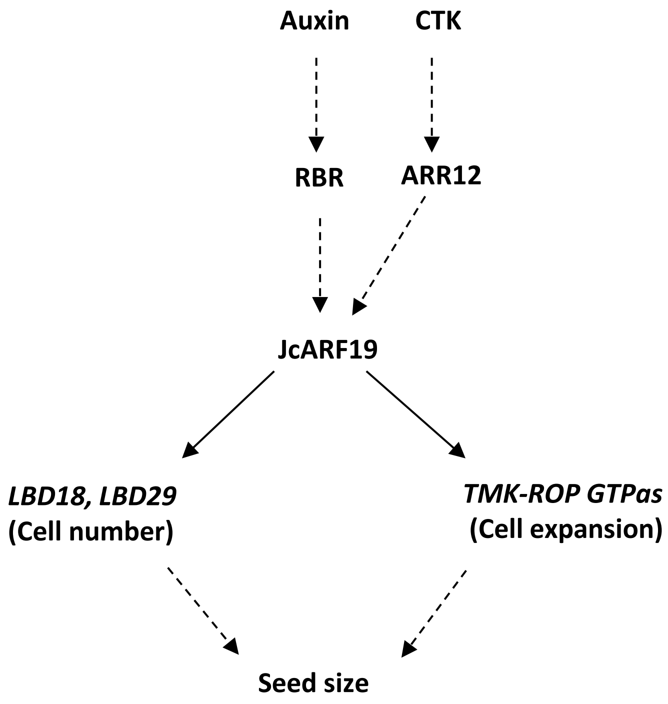

Figure S5 Working model for JcARF19-mediated seed size controlling in *Jatropha*. In this model, the transcription of JcARF19 was activated via RETINOBLASTOMA-RELATED (RBR) protein in auxin signalling and cytokinin-dependent transcription factor ARABIDOPSIS RESPONSE REGULATOR12 (ARR12). JcARF19 is involved in promoting cell differentiation and thus cell number increasing in early stage of seed development by regulating the transcription of *LBD18* and *LBD29.* Meanwhile, JcARF19 is also involved in enlarged cell size by TMK Auxin-Sensing and ROP GTPase signaling complex in middle stage of seed development.
